# Risk factors of Trichomonas vaginalis in women attending central Sexually Transmitted Diseases Clinic Sri Lanka

**DOI:** 10.1186/1742-4690-9-S1-P42

**Published:** 2012-05-25

**Authors:** Sathyadevi Herath, Deepika Fernando, Saman Jayasinge

**Affiliations:** 1 Ministry of Health, Sri Lanka, Colombo, Sri Lanka

## Introduction

*Trichomonas vaginalis* is one of the common infections among women attending Sexually Transmitted Disease Clinics (STD) of Sri Lanka. Yet majority with symptoms don’t attend STD clinics and treated syndromically. In this scenario this study was carried out to identify risk factors of *Trichomonas vaginalis* in women inorder to review treatment by signs and symptoms.

## Methods

Three hundred and fifty new female clinic attendees were recruited. Participants were interviewed on sociodemographic data, sexual history, symptoms, knowledge on STI/HIV, and condom use. Laboratory specimens were collected for theroutinescreening ofSTD diagnosis including Trichomoniasis.

## Results

Mean age of the sample was 32.8 years (SD ± 9.27). More than half (223; 64%) were married. Approximately 53% had completed Grade 10. Almost 76% tested positive being in 21-45 years and 20% of positives were unmarried. Trichomoniasis prevalence was 7.2% (25 out of 346). Pruritus, vaginal discharge and vulvovaginal soreness, were significantly higher amongst positives (P< 0.05 for all). Educated women had higher risk (OR= 3.0; CI=1.28-7.26) of infection. Trichomoniasis was less common among women engaged in sex work (OR= 0.3; 95% CI=.0.14-0.85), reported multiple sexual partners (OR = 0.02; 95% CI= 0.073-0.408) and women reporting extra marital relationship (OR = 0.3%, 95% CI= 0.123-0. 733).

## Conclusions

These findings are the reflection of female clinic attendees involving in commercial sex trade and high use of condoms amongst sex workers. It further emphasizes that the primary prevention activities are widespread especially among MARPs.

Findings also suggest that non use of condoms among women in monogamous relationship may contribute for Trichomoniasis thus HIV acquisition among housewives in Sri Lanka.

